# Supplementary material for: Key mechanistic features of the trade-off between antibody escape and host cell binding in the SARS-CoV-2 Omicron variant spike proteins
Source: EMBO J. 2024 Mar 11;43(8):5. doi: 10.1038/s44318-024-00062-z (PMC11021471; doi:10.1038/s44318-024-00062-z)
Supplement: Supplementary file 1 — Table EV1 [file 44318_2024_62_MOESM1_ESM.docx]

**Table EV1** The immobilization and concentrations statistics of SPR assay to test the binding affinities between hACE2 and RBD or S.

| **Ligand** | **Immobilization quantity (units)** | **Concentrations of hACE2 (nM)** | ***k*_a_ (1/Ms)** | ***k*_d_ (1/s)** | ***K*_D_ (M)** | **Average *K*_D_ (M)** | **SD  (M)** |
| --- | --- | --- | --- | --- | --- | --- | --- |
| BA.4/5 RBD | 664.8 | 200, 100, 50, 25, 12.5 | 5.53*10^4^ | 7.19*10^-4^ | 1.30*10^-8^ | 1.07*10^-8^ | 1.60*10^-9^ |
|  |  |  | 7.40*10^4^ | 7.19*10^-4^ | 9.71*10^-9^ |  |  |
|  |  |  | 7.55*10^4^ | 7.17*10^-4^ | 9.50*10^-9^ |  |  |
| BF.7 RBD | 956.0 | 200, 100, 50, 25, 12.5 | 1.43*10^5^ | 2.20*10^-3^ | 1.53*10^-8^ | 1.30*10^-8^ | 2.38*10^-9^ |
|  |  |  | 1.45*10^5^ | 2.02*10^-3^ | 1.39*10^-8^ |  |  |
|  |  |  | 2.21*10^5^ | 2.14*10^-3^ | 9.70*10^-9^ |  |  |
| BQ.1 RBD | 992.5 | 200, 100, 50, 25, 12.5 | 1.28*10^5^ | 1.35*10^-3^ | 1.06*10^-8^ | 1.03*10^-9^ | 5.58*10^-10^ |
|  |  |  | 1.31*10^5^ | 1.41*10^-3^ | 1.08*10^-8^ |  |  |
|  |  |  | 1.53*10^5^ | 1.46*10^-3^ | 9.53*10^-9^ |  |  |
| BQ.1.1 RBD | 927.6 | 200, 100, 50, 25, 12.5 | 1.23*10^5^ | 1.31*10^-3^ | 1.07*10^-8^ | 1.05*10^-9^ | 4.03*10^-10^ |
|  |  |  | 1.25*10^5^ | 1.35*10^-3^ | 1.08*10^-8^ |  |  |
|  |  |  | 1.42*10^5^ | 1.41*10^-3^ | 9.90*10^-9^ |  |  |
| XBB RBD | 1754.3 | 200, 100, 50, 25, 12.5 | 2.56*10^5^ | 9.77*10^-3^ | 3.82*10^-8^ | 3.72*10^-8^ | 2.13*10^-9^ |
|  |  |  | 2.51*10^5^ | 9.82*10^-3^ | 3.91*10^-8^ |  |  |
|  |  |  | 2.91*10^5^ | 9.95*10^-3^ | 3.42*10^-8^ |  |  |
| XBB.1.5 RBD | 1261.5 | 200, 100, 50, 25, 12.5 | 1.62*10^5^ | 2.24*10^-3^ | 1.39*10^-8^ | 1.39*10^-8^ | 4.08*10^-10^ |
|  |  |  | 1.64*10^5^ | 2.35*10^-3^ | 1.44*10^-8^ |  |  |
|  |  |  | 1.84*10^5^ | 2.46*10^-3^ | 1.34*10^-8^ |  |  |
| XBB RBD | 1226.2 | 200, 100, 50, 25, 12.5 | 2.73*10^5^ | 8.80*10^-3^ | 3.22*10^-8^ | 3.26*10^-8^ | 1.13*10^-9^ |
|  |  |  | 2.79*10^5^ | 8.74*10^-3^ | 3.14*10^-8^ |  |  |
|  |  |  | 2.67*10^5^ | 9.08*10^-3^ | 3.41*10^-8^ |  |  |
| XBB.1.5 RBD | 1440.1 | 200, 100, 50, 25, 12.5 | 2.27*10^5^ | 2.73*10^-3^ | 1.21*10^-8^ | 1.20*10^-8^ | 9.43*10^-11^ |
|  |  |  | 2.31*10^5^ | 2.74*10^-3^ | 1.19*10^-8^ |  |  |
|  |  |  | 2.35*10^5^ | 2.84*10^-3^ | 1.21*10^-8^ |  |  |
| XBB RBD S486F | 1064.2 | 200, 100, 50, 25, 12.5 | 2.28*10^5^ | 1.24*10^-3^ | 5.46*10^-9^ | 5.53*10^-9^ | 1.33*10^-10^ |
|  |  |  | 2.31*10^5^ | 1.25*10^-3^ | 5.42*10^-9^ |  |  |
|  |  |  | 2.36*10^5^ | 1.35*10^-3^ | 5.72*10^-9^ |  |  |
| XBB RBD S486V | 1185.6 | 200, 100, 50, 25, 12.5 | 2.16*10^5^ | 3.80*10^-3^ | 1.75*10^-8^ | 1.83*10^-8^ | 8.04*10^-10^ |
|  |  |  | 2.05*10^5^ | 3.96*10^-3^ | 1.94*10^-8^ |  |  |
|  |  |  | 2.19*10^5^ | 3.93*10^-3^ | 1.80*10^-8^ |  |  |
| BF.7 RBD | 2216.5 | 200, 100, 50, 25, 12.5 | 4.01*10^4^ | 4.70*10^-4^ | 1.17*10^-8^ | 1.03*10^-8^ | 1.04*10^-9^ |
|  |  |  | 5.05*10^4^ | 4.69*10^-4^ | 9.29*10^-9^ |  |  |
|  |  |  | 4.88*10^4^ | 4.77*10^-4^ | 9.77*10^-9^ |  |  |
| BF.7 RBD Q493R | 1874.5 | 200, 100, 50, 25, 12.5 | 5.56*10^4^ | 2.60*10^-4^ | 4.68*10^-9^ | 4.51*10^-9^ | 1.48*10^-10^ |
|  |  |  | 5.93*10^4^ | 2.69*10^-4^ | 4.53*10^-9^ |  |  |
|  |  |  | 6.24*10^4^ | 2.70*10^-4^ | 4.32*10^-9^ |  |  |
| BA.4/5 RBD | 501.0 | 200, 100, 50, 25, 12.5 | 6.52*10^4^ | 7.80*10^-4^ | 1.20*10^-8^ | 1.21*10^-8^ | 1.24*10^-10^ |
|  |  |  | 6.35*10^4^ | 7.66*10^-4^ | 1.21*10^-8^ |  |  |
|  |  |  | 6.31*10^4^ | 7.77*10^-4^ | 1.23*10^-8^ |  |  |
| BA.4/5 RBD Q493R | 506.6 | 200, 100, 50, 25, 12.5 | 9.26*10^4^ | 6.79*10^-3^ | 7.33*10^-8^ | 7.21*10^-8^ | 1.58*10^-9^ |
|  |  |  | 9.78*10^4^ | 7.16*10^-3^ | 7.32*10^-8^ |  |  |
|  |  |  | 1.02*10^3^ | 7.12*10^-3^ | 6.99*10^-8^ |  |  |
| BA.2.75 RBD | 1648.5 | 200, 100, 50, 25, 12.5 | 3.14*10^4^ | 2.57*10^-4^ | 8.18*10^-9^ | 8.21*10^-9^ | 5.88*10^-10^ |
|  |  |  | 3.15*10^4^ | 2.36*10^-4^ | 7.51*10^-9^ |  |  |
|  |  |  | 3.26*10^4^ | 2.92*10^-4^ | 8.95*10^-9^ |  |  |
| BA.2.75 RBD Q493R | 1376.4 | 200, 100, 50, 25, 12.5 | 4.95*10^4^ | 1.56*10^-3^ | 3.16*10^-8^ | 3.15*10^-8^ | 2.62*10^-10^ |
|  |  |  | 4.91*10^4^ | 1.56*10^-3^ | 3.17*10^-8^ |  |  |
|  |  |  | 5.29*10^4^ | 1.65*10^-3^ | 3.11*10^-8^ |  |  |
| BM.1.1.1 RBD | 3715.3 | 200, 100, 50, 25, 12.5 | 3.17*10^4^ | 2.21*10^-3^ | 6.99*10^-8^ | 7.17*10^-8^ | 1.71*10^-9^ |
|  |  |  | 4.24*10^4^ | 3.02*10^-3^ | 7.12*10^-8^ |  |  |
|  |  |  | 3.30*10^4^ | 2.44*10^-3^ | 7.40*10^-8^ |  |  |
| BM.1.1.1 RBD Q493R | 2403.9 | 200, 100, 50, 25, 12.5 | 3.71*10^4^ | 1.07*10^-3^ | 2.89*10^-8^ | 2.84*10^-8^ | 3.86*10^-10^ |
|  |  |  | 3.86*10^4^ | 1.08*10^-3^ | 2.80*10^-8^ |  |  |
|  |  |  | 3.91*10^4^ | 1.10*10^-3^ | 2.82*10^-8^ |  |  |
| BA.4/5 S | 1493.7 | 200, 100, 50, 25, 12.5 | 7.35*10^4^ | 1.10*10^-3^ | 1.49*10^-8^ | 1.44*10^-8^ | 4.11*10^-10^ |
|  |  |  | 7.89*10^4^ | 1.10*10^-3^ | 1.39*10^-8^ |  |  |
|  |  |  | 7.71*10^4^ | 1.10*10^-3^ | 1.43*10^-8^ |  |  |
| BF.7 S | 1358.1 | 200, 100, 50, 25, 12.5 | 7.39*10^4^ | 1.01*10^-3^ | 1.37*10^-8^ | 1.34*10^-8^ | 2.87*10^-10^ |
|  |  |  | 7.85*10^4^ | 1.02*10^-3^ | 1.30*10^-8^ |  |  |
|  |  |  | 7.67*10^4^ | 1.03*10^-3^ | 1.34*10^-8^ |  |  |
| BQ.1 S | 1364.7 | 200, 100, 50, 25, 12.5 | 5.56*10^4^ | 1.11*10^-3^ | 1.99*10^-8^ | 1.91*10^-8^ | 4.08*10^-9^ |
|  |  |  | 5.21*10^4^ | 1.23*10^-3^ | 2.36*10^-8^ |  |  |
|  |  |  | 7.89*10^4^ | 1.08*10^-3^ | 1.37*10^-8^ |  |  |
| BQ.1.1 S | 1220.8 | 200, 100, 50, 25, 12.5 | 6.13*10^4^ | 9.29*10^-4^ | 1.52*10^-8^ | 1.47*10^-8^ | 3.74*10^-10^ |
|  |  |  | 6.55*10^4^ | 9.37*10^-4^ | 1.43*10^-8^ |  |  |
|  |  |  | 6.47*10^4^ | 9.46*10^-4^ | 1.46*10^-8^ |  |  |
| XBB S | 1128.2 | 200, 100, 50, 25, 12.5 | 1.54*10^5^ | 1.77*10^-2^ | 1.15*10^-7^ | 1.24*10^-7^ | 6.18*10^-9^ |
|  |  |  | 8.50*10^4^ | 1.08*10^-2^ | 1.27*10^-7^ |  |  |
|  |  |  | 1.10*10^5^ | 1.41*10^-2^ | 1.29*10^-7^ |  |  |
| XBB.1.5 S | 784.1 | 200, 100, 50, 25, 12.5 | 7.68*10^4^ | 1.94*10^-3^ | 2.53*10^-8^ | 2.60*10^-8^ | 9.43*10^-10^ |
|  |  |  | 7.94*10^4^ | 2.01*10^-3^ | 2.53*10^-8^ |  |  |
|  |  |  | 7.42*10^4^ | 2.02*10^-3^ | 2.73*10^-8^ |  |  |
