# Supplementary material for: Key mechanistic features of the trade-off between antibody escape and host cell binding in the SARS-CoV-2 Omicron variant spike proteins
Source: EMBO J. 2024 Mar 11;43(8):5. doi: 10.1038/s44318-024-00062-z (PMC11021471; doi:10.1038/s44318-024-00062-z)
Supplement: Supplementary file 2 — Table EV2 [file 44318_2024_62_MOESM2_ESM.docx]

**Table EV5** The immobilization and concentrations statistics of SPR assay to test the binding affinities between mAbs or hACE2 and RBD of BA.2, BA.2 F486V, BA.2 R493Q or BA.4/5.

| **Ligand** | **Analyte** | ***k*_a_ (1/Ms)** | ***k*_d_ (1/s)** | ***K*_D_ (M)** | **Average *K*_D_ (M)** | **SD  (M)** |
| --- | --- | --- | --- | --- | --- | --- |
| BD604 | BA.2 RBD | 1.96*10^6^ | 6.84*10^-3^ | 3.49*10^-9^ | 3.68*10^-9^ | 1.39*10^-10^ |
|  |  | 2.23*10^6^ | 8.34*10^-3^ | 3.75*10^-9^ |  |  |
|  |  | 2.08*10^6^ | 7.93*10^-3^ | 3.81*10^-9^ |  |  |
|  | BA.2 RBD F486V | 4.57*10^6^ | 2.19*10^-1^ | 4.80*10^-8^ | 4.66*10^-8^ | 1.34*10^-9^ |
|  |  | 2.44*10^6^ | 1.09*10^-1^ | 4.48*10^-8^ |  |  |
|  |  | 2.60*10^6^ | 1.22*10^-1^ | 4.70*10^-8^ |  |  |
|  | BA.2 RBD R493Q | 1.71*10^6^ | 1.90*10^-2^ | 1.11*10^-8^ | 1.14*10^-8^ | 3.40*10^-10^ |
|  |  | 1.89*10^6^ | 2.25*10^-2^ | 1.19*10^-8^ |  |  |
|  |  | 1.82*10^6^ | 2.05*10^-2^ | 1.13*10^-8^ |  |  |
|  | BA.4/5 RBD | 1.15*10^5^ | 4.61*10^-3^ | 4.03*10^-8^ | 3.97*10^-8^ | 2.81*10^-9^ |
|  |  | 1.32*10^5^ | 4.74*10^-3^ | 3.60*10^-8^ |  |  |
|  |  | 9.51*10^4^ | 4.07*10^-3^ | 4.28*10^-8^ |  |  |
| BD629 | BA.2 RBD | 2.15*10^6^ | 5.56*10^-2^ | 2.59*10^-8^ | 2.84*10^-8^ | 1.80*10^-9^ |
|  |  | 2.40*10^6^ | 7.09*10^-2^ | 2.95*10^-8^ |  |  |
|  |  | 3.64*10^6^ | 1.09*10^-1^ | 2.99*10^-8^ |  |  |
|  | BA.2 RBD F486V | 2.33*10^8^ | 2.91*10^1^ | 1.25*10^-7^ | 1.37*10^-7^ | 1.38*10^-8^ |
|  |  | 5.45*10^5^ | 8.47*10^-2^ | 1.56*10^-7^ |  |  |
|  |  | 1.69*10^6^ | 2.18*10^-1^ | 1.29*10^-7^ |  |  |
|  | BA.2 RBD R493Q | 6.80*10^5^ | 4.05*10^-3^ | 5.95*10^-9^ | 8.52*10^-9^ | 2.28*10^-9^ |
|  |  | 4.31*10^5^ | 4.96*10^-3^ | 1.15*10^-8^ |  |  |
|  |  | 7.48*10^5^ | 6.06*10^-3^ | 8.10*10^-9^ |  |  |
|  | BA.4/5 RBD | 3.68*10^4^ | 3.39*10^-3^ | 9.22*10^-8^ | 9.35*10^-8^ | 2.92*10^-9^ |
|  |  | 3.65*10^4^ | 3.56*10^-3^ | 9.75*10^-8^ |  |  |
|  |  | 3.82*10^4^ | 3.47*10^-3^ | 9.07*10^-8^ |  |  |
| P2C-1F11 | BA.2 RBD | 6.55*10^5^ | 1.21*10^-2^ | 1.85*10^-8^ | 1.81*10^-8^ | 2.02*10^-9^ |
|  |  | 6.08*10^5^ | 9.41*10^-3^ | 1.55*10^-8^ |  |  |
|  |  | 5.82*10^5^ | 1.19*10^-2^ | 2.04*10^-8^ |  |  |
|  | BA.2 RBD F486V | 5.26*10^7^ | 1.71*10^1^ | 3.25*10^-7^ | 3.15*10^-7^ | 6.94*10^-9^ |
|  |  | 4.06*10^5^ | 1.27*10^-1^ | 3.12*10^-7^ |  |  |
|  |  | 3.47*10^5^ | 1.07*10^-1^ | 3.09*10^-7^ |  |  |
|  | BA.2 RBD R493Q | 5.69*10^5^ | 3.12*10^-4^ | 5.49*10^-10^ | 5.87*10^-10^ | 1.11*10^-10^ |
|  |  | 5.41*10^5^ | 4.00*10^-4^ | 7.38*10^-10^ |  |  |
|  |  | 7.61*10^5^ | 3.61*10^-4^ | 4.75*10^-10^ |  |  |
|  | BA.4/5 RBD | 1.13*10^5^ | 5.94*10^-3^ | 5.25*10^-8^ | 5.35*10^-8^ | 1.28*10^-9^ |
|  |  | 1.14*10^5^ | 6.03*10^-3^ | 5.27*10^-8^ |  |  |
|  |  | 1.13*10^5^ | 6.24*10^-3^ | 5.53*10^-8^ |  |  |
| S2K146 | BA.2 RBD | 4.16*10^5^ | 2.80*10^-3^ | 6.72*10^-9^ | 4.59*10^-9^ | 1.55*10^-9^ |
|  |  | 6.23*10^5^ | 1.90*10^-3^ | 3.05*10^-9^ |  |  |
|  |  | 6.11*10^5^ | 2.45*10^-3^ | 4.01*10^-9^ |  |  |
|  | BA.2 RBD F486V | -- | -- | -- | -- | -- |
|  |  | -- | -- | -- |  |  |
|  |  | -- | -- | -- |  |  |
|  | BA.2 RBD R493Q | 2.39*10^6^ | 9.64*10^-7^ | 4.04*10^-13^ | 1.48*10^-12^ | 1.58*10^-12^ |
|  |  | 4.11*10^5^ | 1.53*10^-6^ | 3.72*10^-12^ |  |  |
|  |  | 1.30*10^6^ | 4.10*10^-7^ | 3.17*10^-13^ |  |  |
|  | BA.4/5 RBD | 1.57*10^6^ | 9.65*10^-2^ | 6.14*10^-8^ | 6.06*10^-8^ | 1.04*10^-9^ |
|  |  | 9.69*10^5^ | 5.73*10^-2^ | 5.91*10^-8^ |  |  |
|  |  | 1.02*10^6^ | 6.24*10^-2^ | 6.12*10^-8^ |  |  |
| hACE2 | BA.2 RBD | 1.67*10^5^ | 2.92*10^-3^ | 1.75*10^-8^ | 1.45*10^-8^ | 2.07*10^-9^ |
|  |  | 1.79*10^5^ | 2.32*10^-3^ | 1.30*10^-8^ |  |  |
|  |  | 1.79*10^5^ | 2.36*10^-3^ | 1.32*10^-8^ |  |  |
|  | BA.2 RBD F486V | 2.24*10^5^ | 6.67*10^-3^ | 2.97*10^-8^ | 2.78*10^-8^ | 1.40*10^-9^ |
|  |  | 2.33*10^5^ | 6.16*10^-3^ | 2.65*10^-8^ |  |  |
|  |  | 2.31*10^5^ | 6.30*10^-3^ | 2.72*10^-8^ |  |  |
|  | BA.2 RBD R493Q | 5.94*10^4^ | 4.45*10^-4^ | 7.49*10^-9^ | 5.69*10^-9^ | 1.28*10^-9^ |
|  |  | 6.21*10^4^ | 3.06*10^-4^ | 4.94*10^-9^ |  |  |
|  |  | 6.28*10^4^ | 2.92*10^-4^ | 4.65*10^-9^ |  |  |
|  | BA.4/5 RBD | 1.07*10^5^ | 1.23*10^-3^ | 1.15*10^-8^ | 9.04*10^-9^ | 1.75*10^-9^ |
|  |  | 1.14*10^5^ | 8.84*10^-4^ | 7.78*10^-9^ |  |  |
|  |  | 1.14*10^5^ | 8.92*10^-4^ | 7.82*10^-9^ |  |  |

“--” indicate that the binding affinity is not detected.
