# Supplementary material for: Key mechanistic features of the trade-off between antibody escape and host cell binding in the SARS-CoV-2 Omicron variant spike proteins
Source: EMBO J. 2024 Mar 11;43(8):5. doi: 10.1038/s44318-024-00062-z (PMC11021471; doi:10.1038/s44318-024-00062-z)
Supplement: Supplementary file 3 — Table EV3 [file 44318_2024_62_MOESM3_ESM.docx]

**Table EV2 Cryo-EM data collection, refinement, and validation statistics**

|  | **BQ.1 RBD/hACE2 complex** | **BF.7 RBD/hACE2 complex** | **BQ.1.1 RBD/hACE2 complex** | **XBB RBD/hACE2 complex** | **XBB.1.5 RBD/hACE2/S304 complex** |
| --- | --- | --- | --- | --- | --- |
| **Data collection and processing** |  |  |  |  |  |
| Voltage (kV) | 300 | 300 | 300 | 300 | 300 |
| Electron exposure (e^-^/Å^2^) | 50 | 50 | 50 | 50 | 50 |
| Defocus range (μm) | -1.0 to -2.0 | -1.0 to -2.0 | -1.0 to -2.0 | -1.0 to -2.0 | -1.0 to -2.0 |
| Pixel size (Å) | 0.85 | 0.85 | 0.85 | 0.85 | 0.85 |
| Number of frames collected | 32 | 32 | 32 | 32 | 32 |
| Micrographs Collected (no.) | 214 | 798 | 375 | 297 | 6,597 |
| Symmetry imposed | C1 | C1 | C1 | C1 | C1 |
| Final particles (no.) | 101,822 | 260,759 | 151,609 | 124,538 | 244,183 |
| Map resolution (Å) | 2.71 | 2.47 | 2.69 | 2.80 | 2.91 |
| FSC threshold | 0.143 | 0.143 | 0.143 | 0.143 | 0.143 |
| **Refinement** |  |  |  |  |  |
| Initial model used (PDB code) | 6LZG | 6LZG | 6LZG | 6LZG | 6LZG |
| Map sharpening methods | DeepEMhancer | DeepEMhancer | DeepEMhancer | DeepEMhancer | DeepEMhancer |
| Model composition |  |  |  |  |  |
| Non-hydrogen atoms | 6,559 | 6,559 | 6,558 | 6,538 | 8,281 |
| Protein residues | 791 | 791 | 791 | 791 | 1,017 |
| Ligands | 11 | 11 | 11 | 10 | 11 |
| R.m.s. deviations |  |  |  |  |  |
| Bond lengths (Å) | 0.003 | 0.003 | 0.006 | 0.007 | 0.004 |
| Bond angles (°) | 0.558 | 0.579 | 0.771 | 1.141 | 0.676 |
| Validation |  |  |  |  |  |
| MolProbity Score | 1.54 | 2.03 | 1.72 | 3.04 | 1.84 |
| Clash Score | 5.47 | 5.63 | 5.55 | 16.54 | 7.55 |
| Poor rotamers (%) | 0.43 | 3.89 | 1.59 | 8.20 | 0.45 |
| Ramachandran plot |  |  |  |  |  |
| Favored | 96.32 | 95.93 | 96.06 | 86.66 | 93.56 |
| Allowed | 3.68 | 4.07 | 3.81 | 10.04 | 5.95 |
| Disallowed | 0 | 0 | 0.13 | 3.30 | 0.50 |
