# Supplementary material for: Key mechanistic features of the trade-off between antibody escape and host cell binding in the SARS-CoV-2 Omicron variant spike proteins
Source: EMBO J. 2024 Mar 11;43(8):5. doi: 10.1038/s44318-024-00062-z (PMC11021471; doi:10.1038/s44318-024-00062-z)
Supplement: Supplementary file 4 — Table EV4 [file 44318_2024_62_MOESM4_ESM.docx]

**Table EV3. Data collection and refinement statistics**

|  | Omicron BQ.1.1 | Omicron BF.7 |
| --- | --- | --- |
| **Data collection** |  |  |
| Space group | P22121 | P41212 |
| Cell dimensions |  |  |
| *a*, *b*, *c* (Å) | 123.58, 135.41, 154.48 | 123.27, 137.38, 155.18 |
| α, β, γ (°)  Wavelength (Å) | 90, 90, 90  0.979 | 90.00, 90, 90.00  0.979 |
| Resolution (Å) | 50-3.46(3.58-3.46) | 50-3.40 (3.52-3.40) |
| *R*_merge_ | 0.173 (0.815) | 0.192 (0.988) |
| *R*_pim_ | 0.104 (0.523) | 0 .098 (0.511) |
| CC1/2 | 0.974 (0.501) | 0.991 (0.600) |
| *I* / σ*I* | 5.1 (1.0) | 6.6 (1.1) |
| Completeness (%) | 96.0 (94.9) | 90.7 (91.6) |
| No. reflections | 32701 (3173) | 33045 (3299) |
| Redundancy | 3.4 (3.0) | 4.5 (4.3) |
|  |  |  |
| **Refinement** |  |  |
| Resolution (Å) | 29.75-3.47 | 29.46-3.41 |
| No. reflections | 32660 | 33002 |
| *R*_work_ / *R*_free_ | 0.2115 /0.2691 | 0.2401/0.2901 |
| No. atoms |  |  |
| Protein | 13053 | 13007 |
| Ligand/ion | 2 | 2 |
| Water | 0 | 0 |
| *B*-factors |  |  |
| Protein | 93.0 | 108.0 |
| Ligand/ion | 66.5 | 76.6 |
| Water |  |  |
| R.m.s. deviations |  |  |
| Bond lengths (Å) | 0.003 | 0.004 |
| Bond angles (°) | 0.574 | 0.681 |
| Ramachandran plot |  |  |
| Favored (%) | 94.73 | 9504 |
| Allowed (%) | 5.08 | 4.89 |
| Outliers (%) | 0.19 | 0.06 |

*Values in parentheses are for highest-resolution shell.
