# Supplementary material for: Key mechanistic features of the trade-off between antibody escape and host cell binding in the SARS-CoV-2 Omicron variant spike proteins
Source: EMBO J. 2024 Mar 11;43(8):5. doi: 10.1038/s44318-024-00062-z (PMC11021471; doi:10.1038/s44318-024-00062-z)
Supplement: Supplementary file 5 — Table EV5 [file 44318_2024_62_MOESM5_ESM.docx]

**Table EV4 Amino acid residues comparison of RBDs from the XBB, XBB.1.5, BQ.1, BQ.1.1, BF.7 and BA.4/5 sub-variants interacting with hACE2**

|  | **XBB RBD /hACE2 complex** | **XBB1.5 RBD /hACE2 complex** | **BQ.1 RBD /hACE2 complex** | **BQ.1.1 RBD /hACE2 complex** | **BF.7 RBD/hACE2 complex** |
| --- | --- | --- | --- | --- | --- |
| S19 | A475 (2), G476 (2), N477 (6, **1**) | A475 (1), G476 (2), N477 (8, **2**) | A475 (1), N477 (8) | A475 (1), N477 (4, **1**) | A475 (2), G476 (6), N477 (10) |
| Q24 | A475 (3), G476 (8), N487 (10, **1**) | A475 (5), G476 (3), N477 (1), N487 (6, **1**) | A475 (6), G476 (3), N477 (1), N487 (4, **1**) | A475 (8), G476 (4), N477 (1), N487 (8, **1**) | A475 (6), G476 (3), N487 (6, **1**) |
| T27 | F456 (9), Y473 (1), | F456 (11), Y473(2), | F456 (6), A475 (1), | F456 (9), A475 (1), | F456 (10), A475 (1), |
|  | Y489 (8) | Y475 (1), Y489 (6) | Y489 (4) | Y489 (2) | Y489 (2) |
| F28 | Y489 (6) | Y489 (6) | Y489 (6) | Y489 (6) | Y489 (7) |
| D30 | L455 (3), F456 (8) | L455 (3), F456 (6) | L455 (4), F456 (2) | L455 (5), F456 (5) | F456 (5) |
| K31 | F456 (3), Y489 (5), S490(8, **2**), Q493 (7) | L455 (2), F456 (5), Y489 (7), S490 (1), Q493 (3, **1**) | F456 (5), Y489 (1), F490 (2), Q493 (6, **1**) | L455 (1), F456 (9), Y489 (7), F490 (4, **1**), Q493 (6) | F456 (5), Y489 (9), F490 (3), Q493 (3, **1**) |
| H34 | N417 (1), Y453 (13), L455 (8), Q493 (9), S494 (8) | N417 (1), Y453  (14), L455 (8),  Q493 (10), S494 (9) | Y453 (13), L455 (11), Q493 (15), S494 (6) | Y453 (14, **1**), L455 (8), Q493 (15), S494 (6) | N417 (1), Y453 (10, **1**), L455 (12), Q493 (18), S494 (3) |
| E35 | Q493 (4) | - | - | - | - |
| D38 | Y449 (7, **1**), R498 (5, **1**) | Y449 (11, **1**), Y495 (1), G496 (1), R498 (5, **1**) | Y449 (9, **2**), G496 (2), R498 (8, **2**),  Y501 (1) | Y449 (8, **2**), G495 (1), R498 (8, **1**) | Y449 (9, **1**), R498 (5, **1**) |
| Y41 | R498 (4), T500 (7, **1**), Y501 (14) | R498 (4), T500 (8, **1**), Y501 (13) | R498 (2), T500 (7, **1**), Y501 (14) | R498 (3), T500 (7, **1**), Y501 (12) | R498 (4), T500 (7, **1**), Y501 (14) |
| Q42 | Y449 (3, **1**), R498 (8) | Y449 (1, **1**), R498 (6) | Y449 (2), R498 (5) | G446 (1), Y449 (2, **1**), R498 (11) | Y449 (3, **1**), R498 (5) |
| L79 | - | - | V486 (1) | V486 (1) |  |
| M82 | N487 (5) | P486 (1), N487 (3) | V486 (2), N487(2) | V486 (2), N487(2) | V486 (1), N487(3) |
| Y83 | N487 (7, **1**), Y489 (1) | N487 (7, **1**), Y489 (1) | N487 (1), Y489 (1) | N487 (5, **1**), Y489 (1) | N487 (2), Y489 (1) |
| V316 | - | R86 (1) | - | - | - |
| N330 | T500 (2) | T500 (3) | - | T500 (1) | T500 (4) |
| K353 | Y501 (14), G502 (3, **1**), H505 (20) | R403 (1), Y501 (18), G502 (3, **1**), H505 (23) | Y501 (16), G502 (3, **1**), H505 (17) | R403 (1), Y501 (16), G502 (3, **1**), H505  (21) | R403 (1), Y501 (15), G502 (3, **1**), H505  (24) |
| G354 | Y501 (1), G502 (6, 1), H505 (3) | Y501 (1), G502 (6), H505 (4) | Y501(1), G502 (6), H505 (3) | G502 (5), H505 (3) | G502 (6), H505 (3) |
| D355 | T500 (6) | T500 (7) | T500 (6) | T500 (4) | T500 (7) |
| R357 | T500 (3) | T500 (2) | T500 (3) | T500 (3) | T500 (3) |
| Total | 251, **10** | 252, **10** | 217, **8** | 245, **11** | 242, **8** |

The numbers in parentheses of XBB-RBD, XBB.1.5-RBD, BQ.1-RBD, BQ.1.1-RBD, BF.7-RBD and BA.4/5-RBD residues represent the number of vdw contacts between the indicated residues with hACE2. The numbers with underline suggest numbers of potential H-bonds between the pairs of residues. vdw

contact was analyzed at a cutoff of 4.5 Å and H-bonds were calculated at a cutoff of 3.5 Å
