# Supplementary material for: Key mechanistic features of the trade-off between antibody escape and host cell binding in the SARS-CoV-2 Omicron variant spike proteins
Source: EMBO J. 2024 Mar 11;43(8):5. doi: 10.1038/s44318-024-00062-z (PMC11021471; doi:10.1038/s44318-024-00062-z)
Supplement: Supplementary file 6 — Appendix [file 44318_2024_62_MOESM6_ESM.pdf]

Appendix for: Key mechanistic features of the trade-off between antibody escape and host cell binding in the SARS-CoV-2 Omicron variant spike proteins

Appendix Table of Contents:

Page 3 Appendix Fig S1

Page 4 Appendix Fig S2

Page 5 Appendix Fig S3

Page 6 Appendix Fig S4

Page 7 Appendix Fig S5

Appendix Figures:

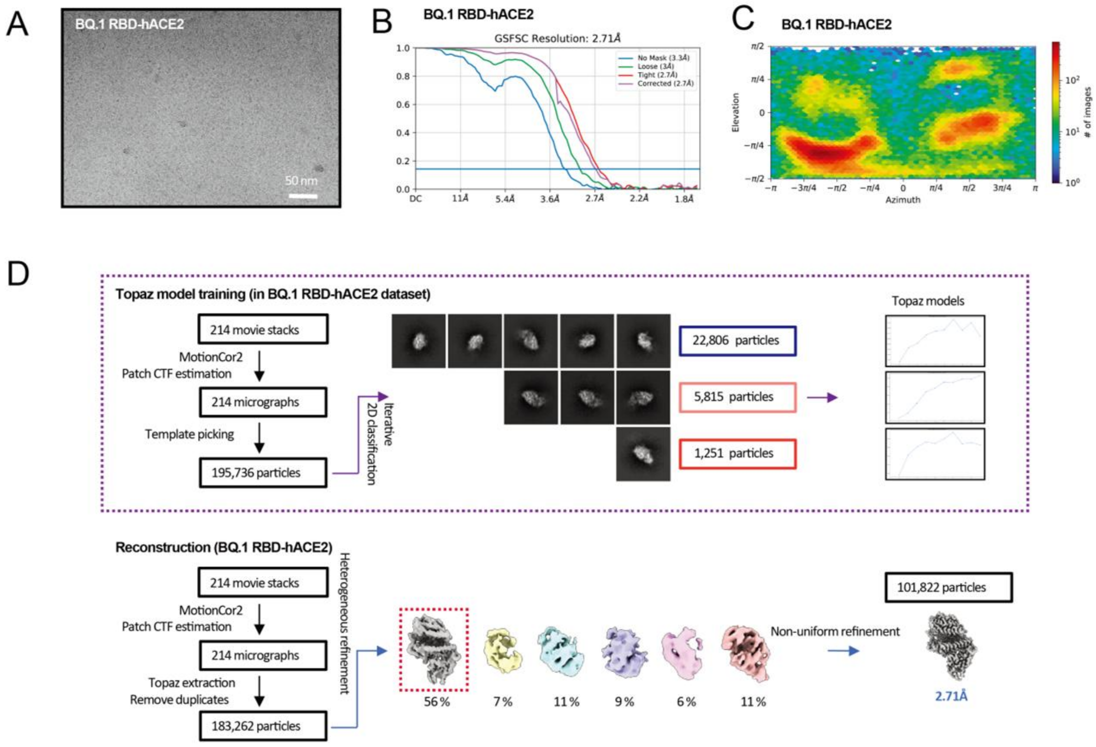

Appendix Fig S1 Flow chart of single-particle analysis of the Omicron BQ.1 RBD/hACE2 complex.

A Representative cryo-EM micrograph of the Omicron BQ.1 RBD/hACE2 complex.

B The FSC curve for the density maps.

C The viewing direction distribution plot.

D A workflow of cryo-EM image processing and reconstruction.

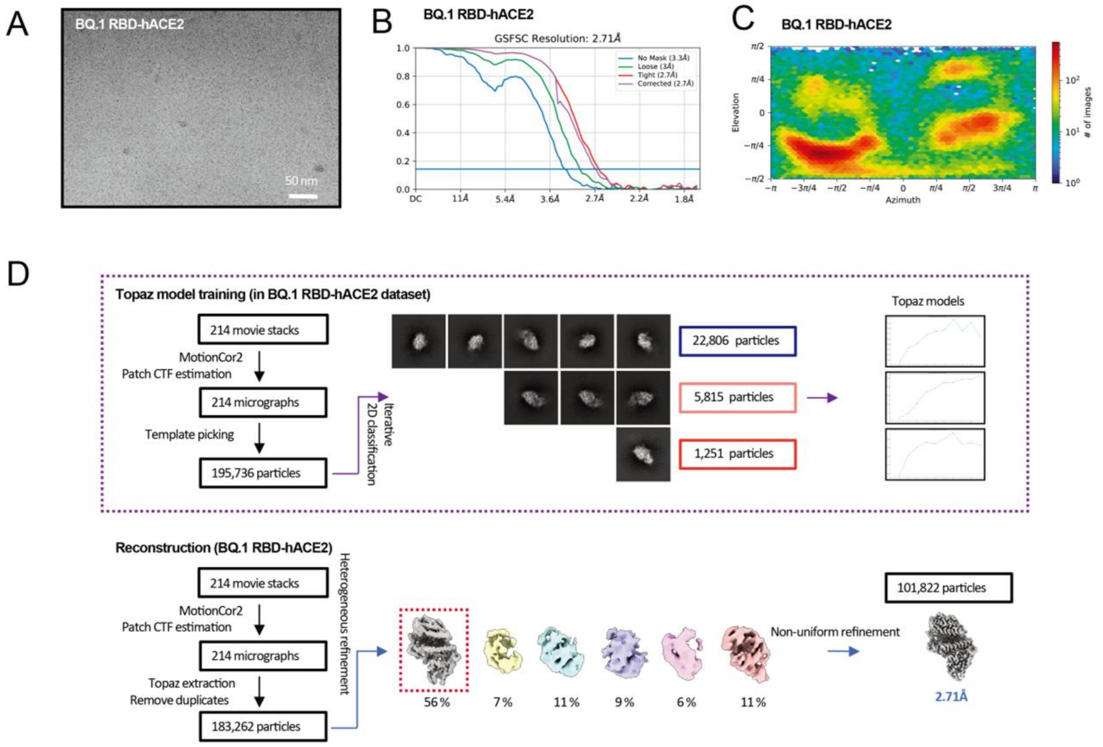

Appendix Fig S2 Flow chart of single-particle analysis of the Omicron XBB RBD/hACE2 complex.

A Representative cryo-EM micrograph of the Omicron XBB RBD/hACE2 complex.

B The FSC curve for the density maps.

C The viewing direction distribution plot.

D A workflow of cryo-EM image processing and reconstruction.

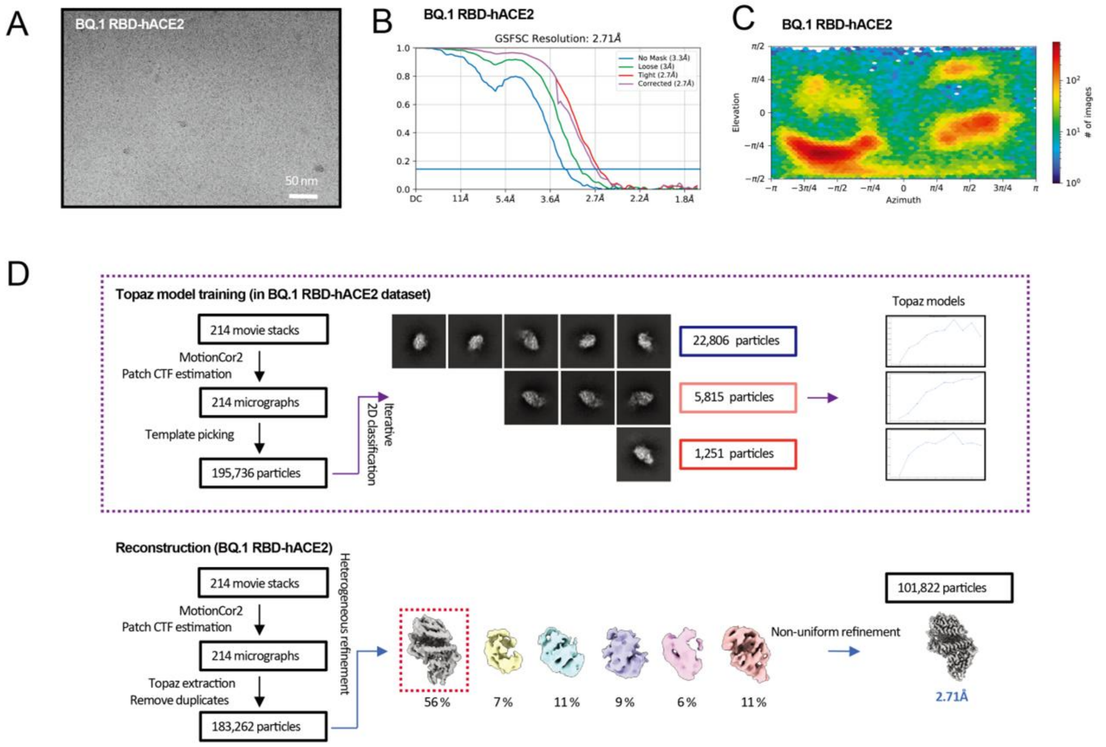

Appendix Fig S3 Flow chart of single-particle analysis of the Omicron BF.7 RBD/hACE2 complex.

A Representative cryo-EM micrograph of the Omicron BF.7 RBD/hACE2 complex.

B The FSC curve for the density maps.

C The viewing direction distribution plot.

D A workflow of cryo-EM image processing and reconstruction.

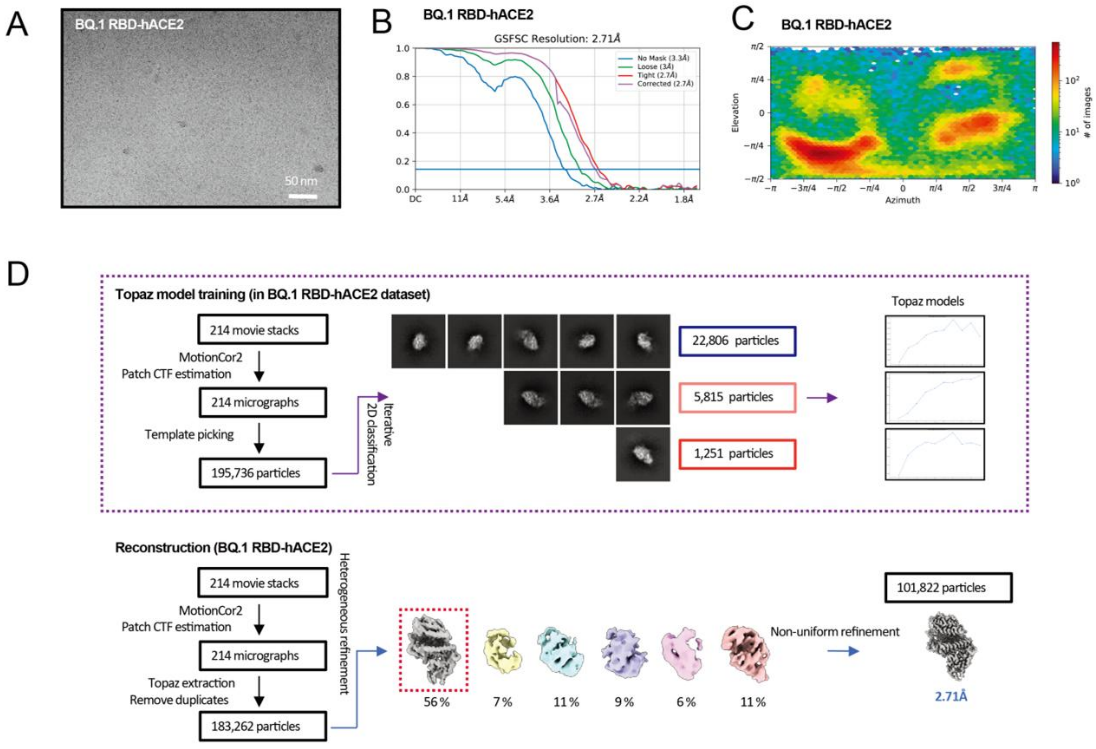

Appendix Fig S4 Flow chart of single-particle analysis of the Omicron BQ.1.1 RBD/hACE2 complex.

A Representative cryo-EM micrograph of the Omicron BQ.1.1 RBD/hACE2 complex.

B The FSC curve for the density maps.

C The viewing direction distribution plot.

D A workflow of cryo-EM image processing and reconstruction.

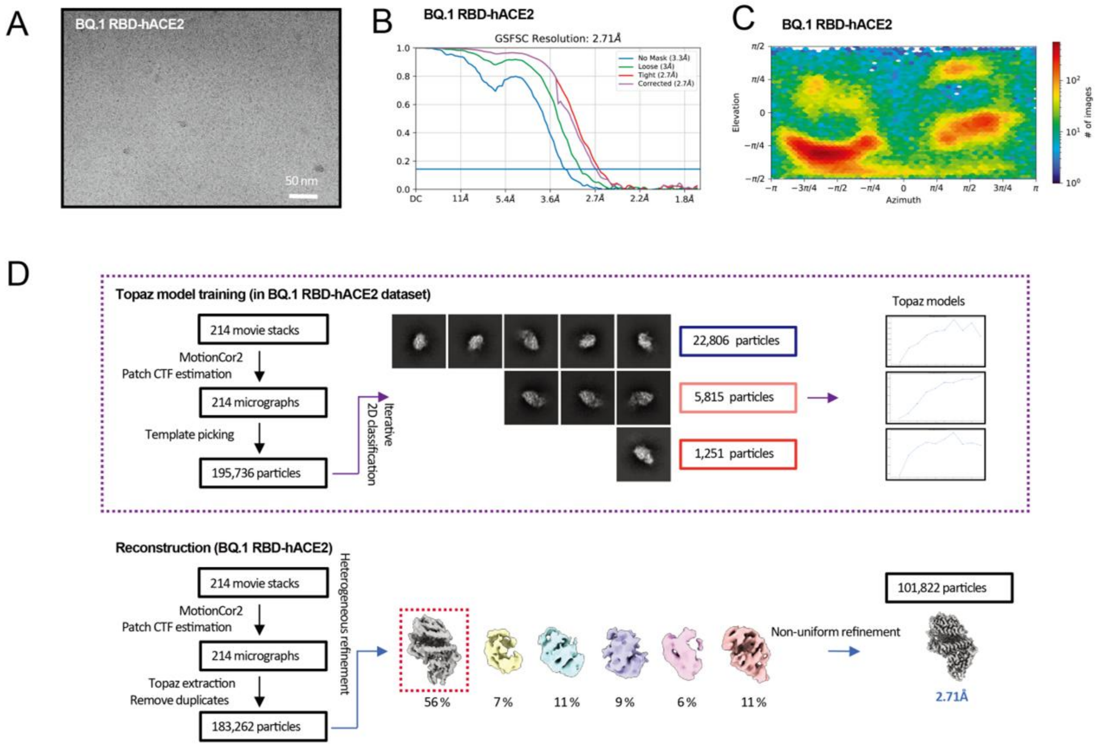

Appendix Fig S5 Flow chart of single-particle analysis of the Omicron XBB.1.5 RBD/hACE2 complex.

A Representative cryo-EM micrograph of the Omicron XBB.1.5 RBD/hACE2 complex.

B The FSC curve for the density maps.

C The viewing direction distribution plot.

D A workflow of cryo-EM image processing and reconstruction.
