# Supplementary material for: Key mechanistic features of the trade-off between antibody escape and host cell binding in the SARS-CoV-2 Omicron variant spike proteins
Source: EMBO J. 2024 Mar 11;43(8):5. doi: 10.1038/s44318-024-00062-z (PMC11021471; doi:10.1038/s44318-024-00062-z)
Supplement: Supplementary file 13 — Expanded View Figures [file 44318_2024_62_MOESM13_ESM.pdf]

Expanded View Figures

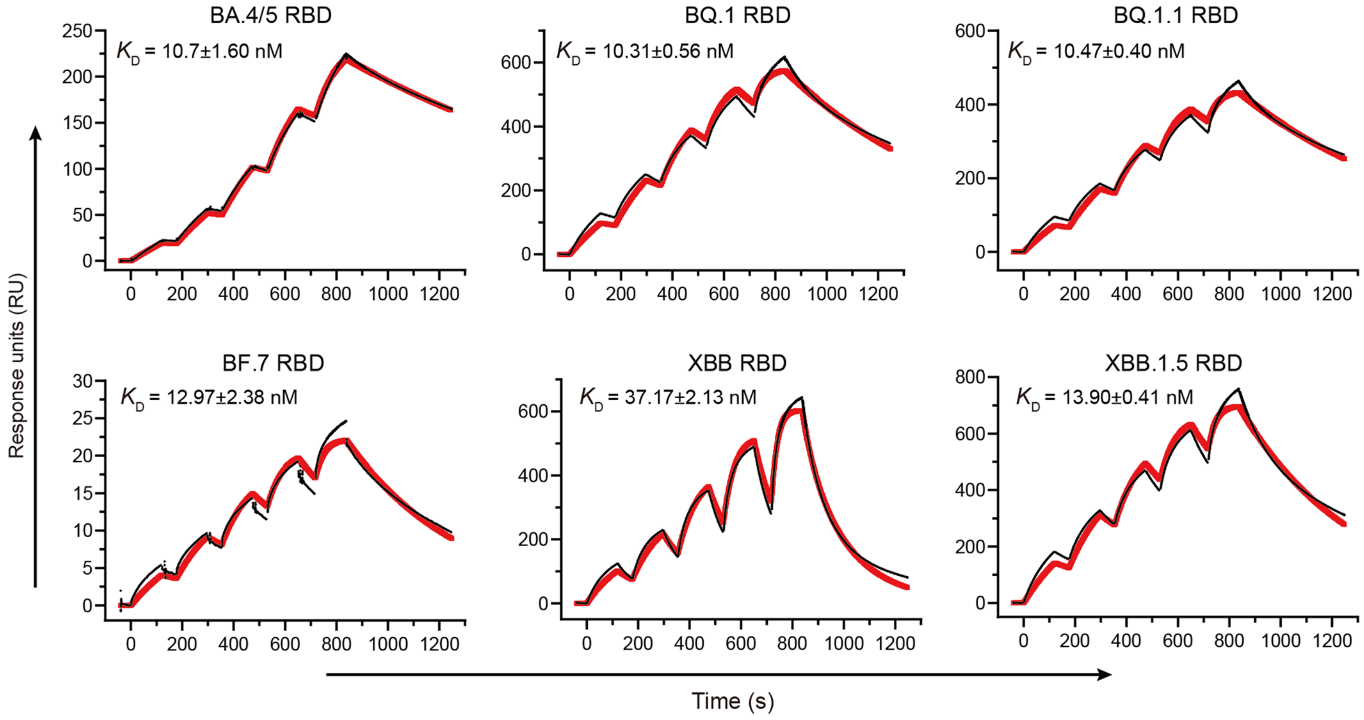

Figure EV1. The SPR curves for the BA.4/5, BQ.1, BQ.1.1, BF.7, XBB, and XBB.1.5 RBD binding to hACE2.

Raw and fitted curves are represented by black and red lines, respectively. Dissociation constant (K_D) indicates mean ± SD from three independent repeats. Source data are available online for this figure.

 © The Author(s)

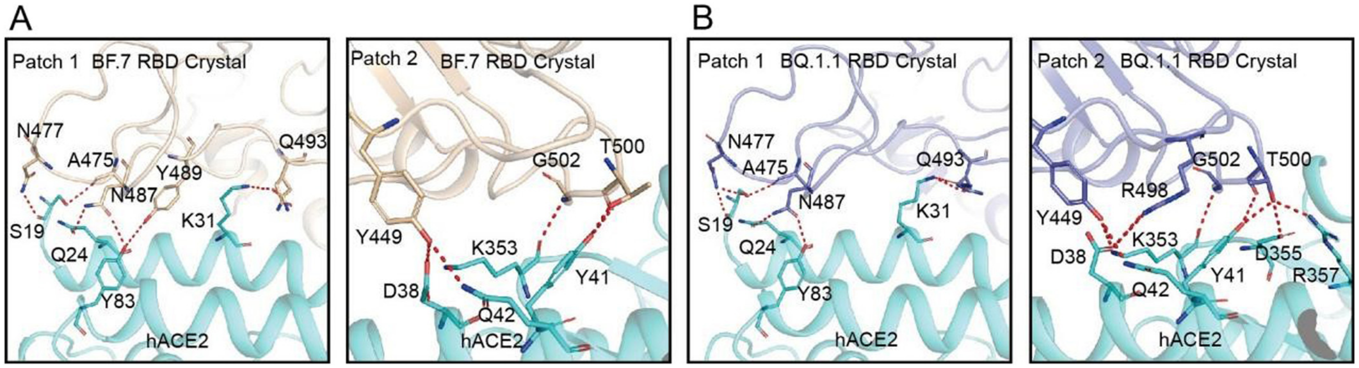

Figure EV2. The crystal structure of BQ.1.1 and BF.7 RBDs in complex with hACE2.

(**A**, **B**) The structures of BF.7 (**A**) and BQ.1.1 (**B**) RBDs in complex with hACE2 are show as cartoons. The residues form H-bonds or salt bridges are shown as sticks. H-bonds and salt bridges are show as red dash lines.

© The Author(s)

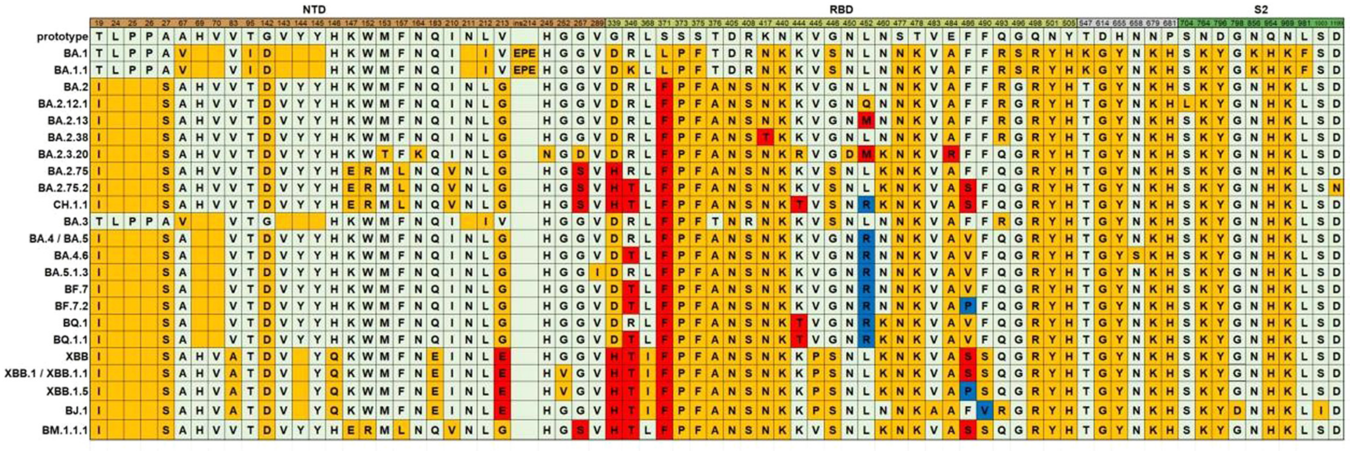

Figure EV3. The statistics of mutations in the S protein of Omicron sub-variants.

Comparing with the S protein of SARS-CoV-2 PT isolate, the mutation sites (including substitutions, deletions and insertions) in the S protein of Omicron sub-variants are highlighted in gold. The third type of amino acid on the same locus is marked in red and the fourth is labeled in blue. Deletion mutations are shown as blank squares.

 © The Author(s)

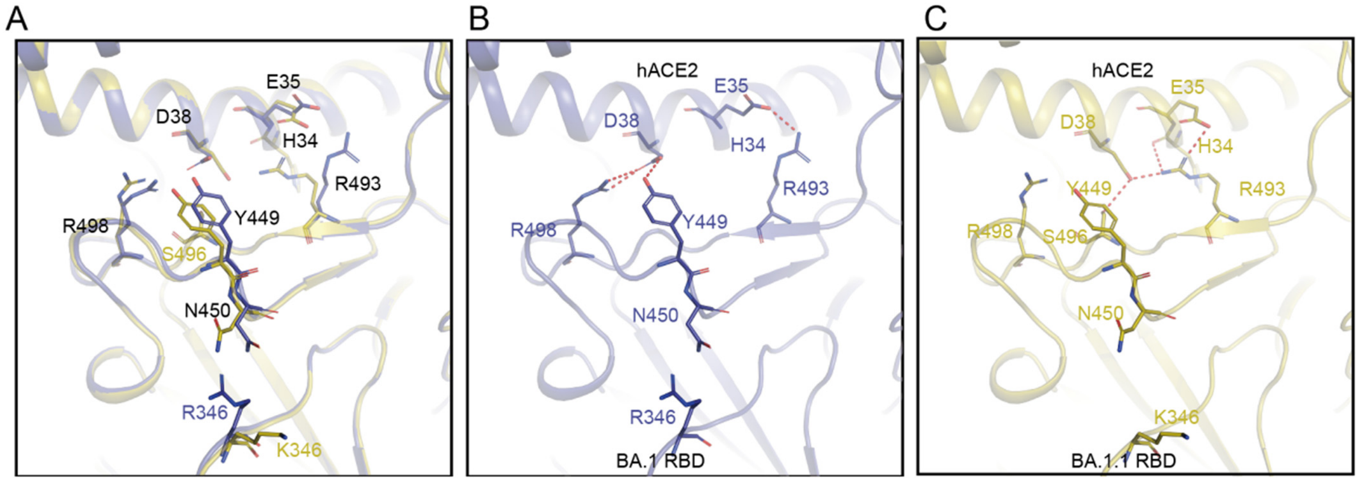

Figure EV4. The structural comparation of BA.1 and BA.1.1 RBDs in complex with hACE2.

(**A**) The structural alignment of BA.1 and BA.1.1 RBDs bound to hACE2. (**B**, **C**) The interaction network of BA.1 RBD/hACE2 complex (**B**) and BA.1.1 RBD/hACE2 complex (**C**). The backbone of the structures are show as cartoon and the key residues are shown as sticks. The H-bonds and salt bridges are shown as red dash lines.
